# The Peptide PbrPSK2 From Phytosulfokine Family Induces Reactive Oxygen Species (ROS) Production to Regulate Pear Pollen Tube Growth

**DOI:** 10.3389/fpls.2020.601993

**Published:** 2020-11-30

**Authors:** Xiaobing Kou, Qian Liu, Yangyang Sun, Peng Wang, Shaoling Zhang, Juyou Wu

**Affiliations:** State Key Laboratory of Crop Genetics and Germplasm Enhancement, Centre of Pear Engineering Technology Research, College of Horticulture, Nanjing Agricultural University, Nanjing, China

**Keywords:** phytosulfokine, pear, WGD events, pollen tube growth, ROS

## Abstract

Phytosulfokines (PSKs) are plant peptide growth factors that participate in multiple biological processes, including cell elongation and immune signaling. However, little is known about PSKs in Rosaceae species. Here, we identified 10 *PSK* genes in pear (*Pyrus bretschneideri*), 11 in apple (*Malus* × *domestica*), four in peach (*Prunus persica*), six in strawberry (*Fragaria vesca*), and five in Chinese plum (*Prunus mume*). In addition, we undertook comparative analysis of the *PSK* gene family in pear and the four other species. Evolutionary analysis indicated that whole genome duplication events (WGD) may have contributed to the expansion of the *PSK* gene family in Rosaceae. Transcriptomes, reverse transcription-PCR and quantitative real-time-PCR analyses were undertaken to demonstrate that *PbrPSK2* is highly expressed in pear pollen. In addition, by adding purified *E. coli*-expressed PbrPSK2 to pollen and using an antisense oligonucleotide approach, we showed that PbrPSK2 can promote pear pollen tube elongation in a dose-dependent manner. Furthermore, PbrPSK2 was found to mediate the production of reactive oxygen species to regulate pear pollen tube growth.

## Introduction

Polypeptide signals play essential roles in many aspects of plant life including growth, development, reproduction and immunity (Story, 1991; Pearce et al., 2001; Peng et al., 2002; Ryan et al., 2002). For example, CLE (CLV3/ESR-related) peptides, identified as intercellular signaling molecules, could regulate cell differentiation and division in plant development (Fiers et al., 2007; Araya et al., 2014). RGF (root meristem growth factor) is a secreted peptide involved in the maintenance of root stem cell niche (Mari et al., 2010; Matsubayashi, 2013). Classified as secreted peptides, IDA (INFLORESCENCE DEFICIENT IN ABSCISSION) are characterized to play important roles in petal abscission (Melinka, 2003; Estornell et al., 2015). PLS (POLARIS) peptide family is identified to be involved in longitudinal cell expansion and increased radial expansion (Topping and Lindsey, 1997). SP11/SCR (S-locus protein 11 or S-locus Cys-rich) is a male determinant, involved in process of *Brassica* self-incompatibility (Shiba et al., 2001; Sato et al., 2004; Fujimoto et al., 2006). Similarly, AtLURE1 is a defensin-like peptide that functions in attracting pollen tubes into the embryo sac in *arabidopsis* (Okuda et al., 2009). SCA (Stigma/stylar cysteine-rich adhesion) belongs to lipid transfer proteins and is involved in adhesion of pollen tube to the extracellular matrix of female tissues (Mollet et al., 2000). RALFs (Rapid alkalization factors) are a group of cysteine-rich peptides (CRPs) regulating pollen tube growth and burst in plant reproduction (Wu et al., 2000; Ge et al., 2017). In addition, phytosulfokine (PSK) is a plant peptide family, plays a role in plant cell growth, and thus employed as the research objective in our study.

Synthesized from 80 to 120 amino acid prepropeptides, PSK is a small peptide containing an N-terminal signal peptide sequence and a C-terminal PSK sequence (Yang et al., 2001). PSK was firstly identified as a key regulator for inducing division in cell cultures at low density (Stuart and Street, 1969). Since the first *PSK* gene was characterized, more members of the *PSK* gene family have been identified in various plant species. For example, *PSK* genes are ubiquitously expressed in *Arabidopsis*, where the peptides are encoded by a small gene family containing six members (Lorbiecke and Sauter, 2002). Moreover, seven *PSK* genes are predicted to exist in the rice (*Oryza sativa*) genome (Yang et al., 1999). Nevertheless, PSKs those in Rosaceae have yet to be characterized and remain unstudied.

Since identified as a signaling molecule, PSKs have been verified functions in plant growth and development (Yamakawa et al., 1998; Yang et al., 1999; Kim et al., 2006). For example, PSK can induce the proliferation of asparagus and rice cells in low-density cell cultures (Matsubayashi et al., 1996). PSKs also act as signaling molecules of root elongation involving in growth regulation. For instance, AtPSK1 affects root elongation primarily via control of mature cell size in *Arabidopsis* (Kutschmar et al., 2009). PSK promotes root growth by repressing expression of pectin methylesterase inhibitor (PMEI) genes in *Medicago truncatula* (Yu et al., 2020). In *Cunninghamia lanceolate*, PSK had been shown to promote primary root growth and adventitious root formation (Wu et al., 2019). Additionally, PSK acts as a growth factor to stimulate somatic embryogenesis in *carrot* (Hanai et al., 2000). PSK interacts with phytosulfokine receptor 1 (PSKR1) to participate in the regulation of hypocotyl length and cell expansion in *Arabidopsis* (Stührwohldt et al., 2011). Known as a significant process that limits crop yield, the drought-induced premature abscission of flowers and fruits is identified to be regulated by PSKs (Reichardt et al., 2020). Furthermore, PSK acts as a signal molecule in the fertilization of female tissues. In tobacco (*Nicotiana tabacum* L.), PSK could promote pollen germination in a dose–dependent manner (Chen et al., 2000). In maize (*Zea mays* L.), two *PSK* precursor genes were shown to be specifically expressed in male and female gametophytes (Lorbiecke et al., 2005). In *Arabidopsis*, PSK peptide signaling (*AtPSK2*) participates in the processes of pollen tube growth and funicular pollen tube guidance (Stührwohldt et al., 2015), indicating that PSK plays important roles in plant reproduction. In addition, crucial regulatory roles for PSK in plant immunity have been confirmed in some *Arabidopsis* cultivars with enhanced plant immunity and resistance (Yamakawa et al., 1999; Igarashi et al., 2012). In Tomato (*Solanum lycopersicum*), PSK peptide Initiates auxin-dependent immunity through cytosolic Ca^2+^ signaling (Zhang et al., 2018). Nevertheless, functional investigations of PSKs have been limited in model plants and the roles of these molecules are poorly understood in the Rosaceae.

Pear is one of the most economically important fruit crops world-wide. The pear genome has recently been reported (Wu et al., 2013), which enables the comprehensive analysis of the *PSK* gene family in pear. In the present study, we conducted a comprehensive analysis of the *PSK* gene family in pear and four other Rosaceae species. In addition, we identified a *PSK* gene in pear, *PbrPSK2*, which can regulate pollen tube growth. These results provide valuable information for future functional studies of PSK in pear.

## Materials and Methods

### Identification of *PSK* Genes in Rosaceae

To identify potential members of the *PSK* gene family in Rosaceae, we performed multiple database searches. The PSK domain PF06404, downloaded from Pfam^1^, was used as a query to perform BLAST searches in HMMER3 software against pear and other Rosaceae genome databases. Apple, peach and strawberry PSK protein sequences were downloaded from the GDR^2^ and Phytozome v.9.1^3^ (Goodstein et al., 2012). Pear PSK protein sequences were downloaded from the pear genome project database^4^ (Wu et al., 2013). Chinese plum PSK protein sequences were downloaded from the *Prunus mume* Genome Project^5^ (Zhang et al., 2012). All *PSK* genes with expected *E*-values of < 0.001 were collected. Candidate sequences were then examined, and SMART^6^ was used to confirm the candidate *PSK* genes.

In addition, EXPASY^7^ was used to compute the theoretical isoelectric point and molecular weight values from the amino acid sequences of the *PSK* genes.

### Rosaceae PSK Domain Sequence Analysis

To determine the level of sequence conservation and functional homology of PSK in pear, apple, peach, strawberry and Chinese plum, multiple sequence alignment of partial selected candidate PSK domains was carried out using ClustalX^8^ in SMS^9^. The PSK domain was acquired from Pfam^10^.

### Phylogenetic Analysis

The complete PSK protein sequence was used to perform multiple sequence alignments using ClustalX (see footnote) with default parameters (Larkin et al., 2007). The neighbor-joining method was used to construct a phylogenetic tree with MEGA 7.0 from the full-length protein sequences of PSK in five Rosaceae species. The phylogenetic trees were presented using EVOLVIEW^11^ (He et al., 2016) and the reliability was tested using the bootstrap method with 1,000 replicates.

### Conserved Motif and Gene Structure Analysis

In order to examine the relationships involved in the structural evolution of *PSK* genes, we compared the gene structures and motifs of individual *PSK* genes in pear, apple, peach, strawberry and Chinese plum. The motifs were constructed online in Multiple Expectation Maximization for Motif Elicitation (MEME)^12^ using the full-length amino acid sequences of all Rosaceae PSK proteins (Bailey et al., 2006). Exon-intron structural information for the *PSK* genes was obtained from the pear, apple, peach, strawberry, and Chinese plum genome project. The motifs and gene structures of the *PSK* genes were redrawn using TBtools (Chen et al., 2018).

### Synteny Analysis of *PSK* Genes

Information on the synteny relationships among pear, apple, peach, strawberry and Chinese plum were obtained from the pear, apple, peach, strawberry, and Chinese plum genome project, respectively (Velasco et al., 2010; Shulaev et al., 2011; Zhang et al., 2012; Verde et al., 2013; Wu et al., 2013), and the orthologous and paralogous relationships among the five species were plotted using the circos project^13^ (Krzywinski et al., 2009). MCScanX was further used to identify whole-genome duplication/segmental, tandem, proximal and dispersed duplications in the PSK gene family (Wang et al., 2012).

### Calculating the Ka and Ks Values for the *PSK* Gene Family

The Ka and Ks substitution rates of the syntenic gene pairs were annotated using MCScanX downstream analysis tools. KaKs_Calculator 2.0 was used to determine Ka and Ks (Wang et al., 2012).

### Gene Inhibition by Antisense Oligodeoxynucleotides (ODN)

The construction of *PbrPSK2* mRNA was predicted using the RNA fold Web Server^14^. The candidate as-ODN sequence was evaluated in DNAMAN and SnapGene. The as-ODN sequence was synthesized from phosphorothioate and purified by high-performance liquid chromatography. Pollen was pre-cultured in liquid medium for 45 min at 25°C, 120 rpm. The culture contained: 0.03% Ca (NO_3_)_2_⋅4H_2_O, 0.01% H_3_BO_3_, 10% sucrose and 0.58% 2-(N-morpholino) ethanesulfonic acid hydrate (MES) at pH 6.2 (adjusted with Tris). The PbrPSK2-asODN primers were mixed with Lipofectamine^TM^ 2000 Transfection Reagent (11668027, Thermo Fisher Scientific, Shanghai) and incubated for 15 min before being added into the pre-cultured pollen. The pollen tubes were kept in the culture for 1.5 h and visualized with a Nikon Eclipse E100 microscope. The lengths of the pollen tubes were measured with IPWin32 software. Finally, pollen samples were centrifuged, the supernatant removed and the precipitate kept. Then, samples were preserved by freezing in liquid nitrogen and stored at −80°C. All materials were harvested for the extraction of total RNA. Quantitative real-time (qRT) PCR was used to detect the expression of *PbrPSK2*. The primers used for this assay are listed in Supplementary Table S4.

### Expression and Purification of PbrPSK2 Protein From *E. coli*

A DNA fragment encoding PbrPSK2 was cloned into the pCold TF vector and expressed in *E. coli* strain BL21. A single positive clone was cultured in medium containing 100 μg/mL ampicillin at 25°C and 220 rpm for 16 h and then transferred to fresh medium at a dilution of 1/100. When the OD600 of the culture reached 0.6–1.2, IPTG was added to induce recombinant protein expression at 15°C and 220 rpm for 24 h. The expression of the recombinant protein was identified by SDS-PAGE. Finally, the purified protein was dialyzed (Spectra/por^®^ membrane, molecular cutoff 2,000–3,000) against 1 L of pollen medium at 4°C for 24 h and stored at -80°C. The primers used for this assay are listed in Supplementary Table S4.

### Nitroblue Tetrazolium (NBT) Assay for the Detection of Reactive Oxygen Species

To detect a change of reactive oxygen species (ROS) in pollen tube tips after PbrPSK2 treatment, pear pollen was pre-cultured in liquid medium at 25°C and 120 rpm for 45 min and then treated with recombinant PbrPSK2 protein. As a negative control, DPI (diphenylene iodonium) and Mn-TMPP [Manganese (III) 5,10,15,20-tetra (4-pyridyl)-21H,23H-porphine] were used to treat the pre-cultured pollen tubes. The pollen was cultured for another 1.5 h. Finally, the pollen tubes were stained with NBT (1 mg/mL) for 5 min. The change in ROS content at pollen tube tips was visualized with a Nikon Eclipse E100 microscope. More than 50 pollen tubes were detected in each treatment, with three repeats.

### Expression Analyses of *PSKs* in Five Pear Tissues

RT-PCR analysis was used to study the expression of PSK genes in five types of tissue, including young root, leave, stem, pollen and pistil from the pear “Dangshansuli.” Total RNA was extracted using a Plant Total RNA Isolation Kit (FOREGENE, Chengdu, China) and genomic DNA contamination was removed by Dnase I. RT-PCR was carried out according to the manufacturer’s instructions. The PCR temperature scheme was regulated according to the oligonucleotide primers employed for the experiments. The RT-PCR process was as follows: 3 min at 94°C, 30 cycles of 30 s at 94°C, 30 s at 60°C, 1 min at 72°C and 10 min of extension at 72°C. All PCR experiments were repeated three times to confirm the reproducibility of the results.

### Analyses of *PbrPSKs* Expression During Pollen Tube Growth

Quantitative RT-PCR was used to analyze the expression of *PbrPSK* genes in pollen during the developmental stages of the pear “Dangshansuli.” Primer 5.0 was used to design the unique primers according to the *PbrPSK* gene sequences. Neither primer dimers nor unexpected products were found and primers were diluted sixfold. qRT-PCR was performed on LightCycler-480 Detection System (Roche, Penzberg, Germany) using AceQ^®^ qPCR SYBR^®^ Green Master Mix (Vazyme, Nanjing, China), according to the manufacturer’s protocol. Reactions were prepared in a total volume of 20 μL containing: 10 μL of AceQ^®^ qPCR SYBR^®^ Green Master Mix, 5 μL of nuclease-free water, 2.5 μL of each diluted primer and 0.1 μL of cDNA. The qRT-PCR began with 5 min at 95°C, followed by 45 cycles at 95°C for 3 s, 60°C for 10 s and 30 s of extension at 72°C. *PbrTUB* was used as reference gene and the relative expression levels were calculated using the 2-ΔΔCt method. All RNA extraction and cDNA synthesis experiments for all samples were performed with three biological and technical replicates.

## Results

### Identification and Bioinformation of *PSK* Genes in the Rosaceae

A Hidden Markov Model search (HMM search) using “Phytosulfokine precursor protein” domain (Pfam: PF06404) was carried out in five Rosaceae genome databases and SMART was used to confirm the candidate *PSK* genes (Eddy, 2011). Protein sequences lacking the PSK domain or with *E*-values > 1e-15 were removed. As a result, 36 candidate *PSK* genes were surveyed in our study. A total of 10 *PSK* genes were identified in pear (*PbrPSKs*), 11 in apple (*MdPSK*s), four in peach (*PpPSKs*), six in strawberry (*FvPSKs*), and five in Chinese plum (*PmPSKs*). The *PbrPSK* genes were distributed unevenly through the genome, with two of the genes located on chromosome 15. Similar to the *PbrPSK* genes, the distribution of *PSK* genes in the other four Rosaceae genomes was also random. We determined that *PbrPSK4* (Chr1:2370098-2370610) and *PbrPSK5* (Chr17: 19789236-19789748) have the same CDS length, isoelectric point and molecular weight (Supplementary Figure S3 and Table 1), and further studies have found that these two genes are, in fact, the same gene located on different chromosomes.

**TABLE 1:** Characteristics of the PSK proteins.

Gene name	Gene ID	Chr locous	Genomic position	CDS length (bp)	Protein length (aa)	PI	MW (kDa)	
PbrPSK1	Pbr033759.1	15	30125362–30126069	249	82	5.05	9	WGD
PbrPSK2	Pbr019776.1	15	7007426–7007908	255	84	5.2	9.3	WGD
PbrPSK3	Pbr001074.1	2	12193941–12194510	255	84	4.85	9.3	WGD
PbrPSK4	Pbr029236.1	1	2370098–2370610	258	85	6.9	9.6	WGD
PbrPSK5	Pbr017982.1	17	19789236–19789748	258	85	6.9	9.6	WGD
PbrPSK6	Pbr030039.1	13	4052766–4053325	306	101	4.64	10.8	WGD
PbrPSK7	Pbr025638.1	10	16763000–16763383	258	85	5.61	9.4	dispersed
PbrPSK8	Pbr005499.1	12	20880364–20881482	249	82	6.05	9.6	WGD
PbrPSK9	Pbr004808.1	3	13115592–13116225	285	94	5.06	10.5	WGD
PbrPSK10	Pbr016802.1	14	3257529–3257919	240	79	4.92	9	WGD
MdPSK1	MD02G1189200	14	17279050–17279779	303	100	4.90	11.1	WGD
MdPSK2	MD15G1066300	15	4612903–4613689	255	84	5.82	9.6	WGD
MdPSK3	MD15G1300000	15	28529252–28529832	249	82	4.85	8.9	WGD
MdPSK4	MD05G1105500	5	21872939–21873522	261	86	4.97	9.3	WGD
MdPSK5	MD13G1053700	13	3785657–3786239	306	101	4.48	10.8	
MdPSK6	MD10G1110000	10	18087718–18088297	258	85	4.68	9.3	WGD
MdPSK7	MD08G1079100	8	6590799–6591412	258	85	7.84	95	WGD
MdPSK8	MD11G1157500	11	15199717–15200525	285	94	5.87	10.6	WGD
MdPSK9	MD14G1016200	14	1461086–1462014	249	82	5.19	9.4	WGD
MdPSK10	MD12G1018200	12	1740431–1741485	249	82	6.71	9.6	WGD
MdPSK11	MD03G1134700	3	13446978–13448023	285	94	4.61	10.4	WGD
PpPSK1	Prupe.6G217500.1	6	22440310–22441230	246	81	4.85	8.9	WGD
PpPSK2	Prupe.8G150200.1	8	16361784–16362457	255	84	4.7	9.3	WGD
PpPSK3	Prupe.7G116900.1	7	14189550–14190256	237	78	5.09	9.1	
PpPSK4	Prupe.1G420000.1	1	36347487–36348242	255	84	5.21	9.3	WGD
FvPSK1	FvH4_1g18100.1	1	10519153–10520025	192	63	10.18	6.8	
FvPSK2	FvH4_6g17920.1	6	11755315–11755987	240	79	5.36	9.2	Dispersed
FvPSK3	FvH4_2g32750.1	2	17924199–17924836	324	107	6.63	12	Dispersed
FvPSK4	FvH4_2g03340.1	2	24747841–24748458	261	86	4.88	9.6	Proximal
FvPSK5	FvH4_2g03370.1	2	2656597–2657767	354	117	4.66	13	Proximal
FvPSK6	FvH4_3g29900.1	3	23002017–23003111	285	94	4.9	10.5	Dispersed
PmPSK1	Pm021096	6	6679335–6679722	255	84	5.05	9.2	WGD
PmPSK2	Pm002355	1	18913407–18913747	234	77	4.83	8.5	WGD
PmPSK3	Pm005428	2	10969139–10969477	252	83	5.21	9.2	WGD
PmPSK4	Pm001229	1	8232164–8232742	291	96	4.58	10.6	WGD
PmPSK5	Pm026233	8	8630210–8630575	237	78	5.69	9.1	WGD

The physical and chemical characteristics of the *PSK* gene family are shown in Table 1. The protein sequence lengths varied from 63 to 117 amino acids, but most were between 80 and 90 amino acids long. The values of the isoelectric point were between 4.48 and 10.18 and the molecular weights of the proteins were between 6.8 and 13 kDa.

### Phylogenetic Analysis and Structural Characterization of the *PSK* Family

**FIGURE 1:**
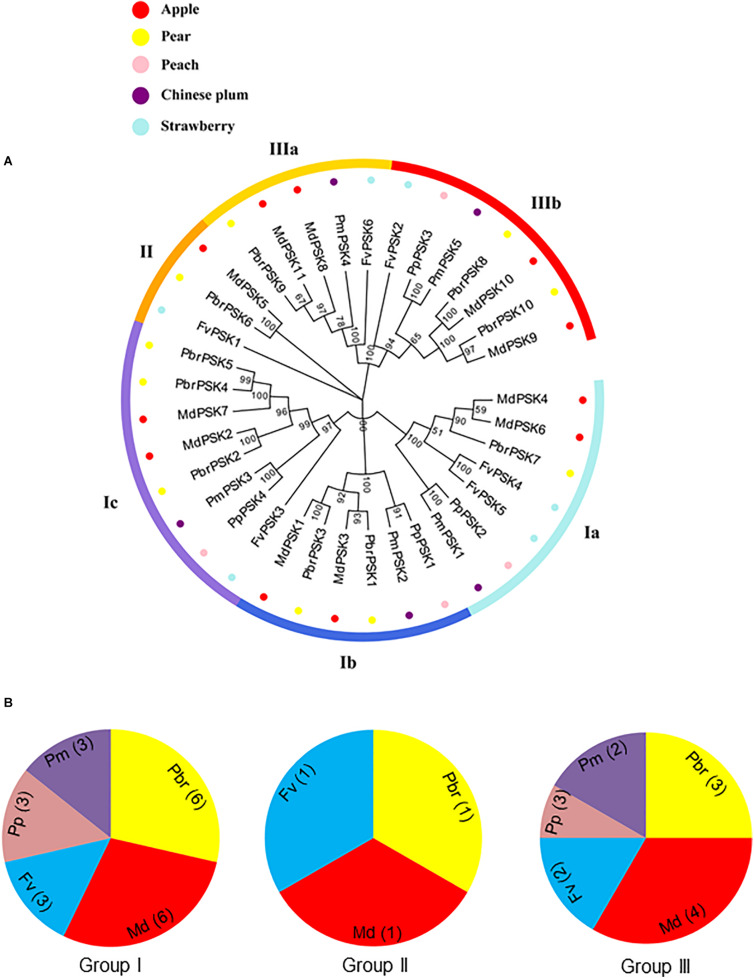
Phylogenetic tree of PSK genes in five Rosaceae species. **(A)** Complete amino acid sequences of PSK proteins identified in five Rosaceae species (pear, apple, peach, strawberry, and Chinese plum) were aligned using Clustal X. The phylogenetic tree was constructed using MEGA7.0 program by the neighbor-joining (NJ) method. A bootstrap test was set as 1,000 replications to test the confidence of the tree. **(B)** The numbers of PSK genes from Rosaceae in group I, II, and III.

In order to examine the evolutionary relationships of *PSK* genes between pear and the other Rosaceae species (apple, peach, strawberry, and Chinese plum), we constructed a neighbor-joining phylogenetic tree in MAGA 7.0 using the full-length protein sequences of 10 PbrPSKs from pear, 11 MdPSKs from apple, four PpPSKs from peach, six FvPSKs from strawberry and five PmPSKs from Chinese plum. According to the phylogenetic tree (Figure 1A), the PSK family members were divided into three major groups (Groups I-III). Interestingly, GroupIIconstituted the smallest branch, with only three members. In addition, GroupIpossessed the largest number of PSK members, with six *PSK* genes in pear (Figure 1B).

**FIGURE 2:**
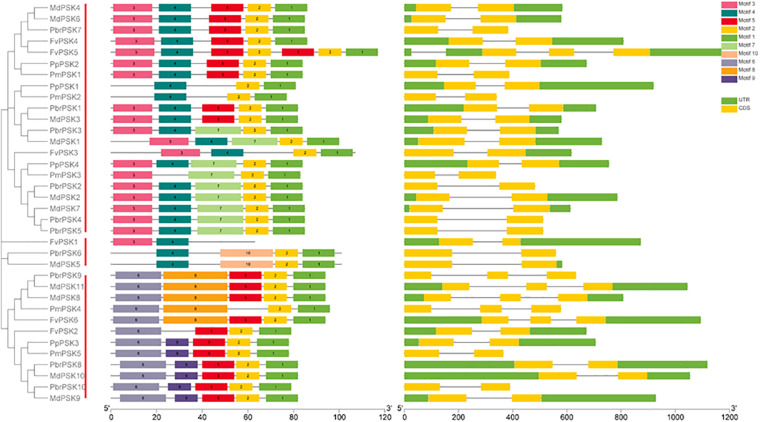
Gene structure and the conserved protein motifs in 36 *PSK* genes from five Rosaceae species. Analysis with MEME to investigate 10 conserved motifs in PSK proteins. Ten motifs (1–10) were identified and are indicated by different colors. Introns and exons are represented by black lines and yellow boxes, respectively. The number indicates the phases of the corresponding introns. Each section of bar represents 0.2 kb.

To investigate the protein structures of PSK in the five Rosaceae species studied, 10 conserved motifs were constructed using the MEME website. From Group I, only PmPSK3 is missing motif 4 and FvPSK5 contains two copies of motif 5, whereas motif 10 only exists in MdPSK5 and PbrPSK6. We also found that, bar FvPSK1, motifs 1 and 2 (containing YIYTQ motif) are present throughout the PSK family in the Rosaceae indicating that they may be conserved among this gene family (Figure 2 and Supplementary Figure S4).

The introns and exons of *PSK* genes were established using GSDS 2.0 (Hu et al., 2015), giving similar results for the motif and gene structures, which were conserved within the same branch. According to the predicted structures, all *PSK* genes have two to three exons with relatively conserved arrangements and similar size.

### Evolutionary Pattern Analysis of *PSK*s

**FIGURE 3:**
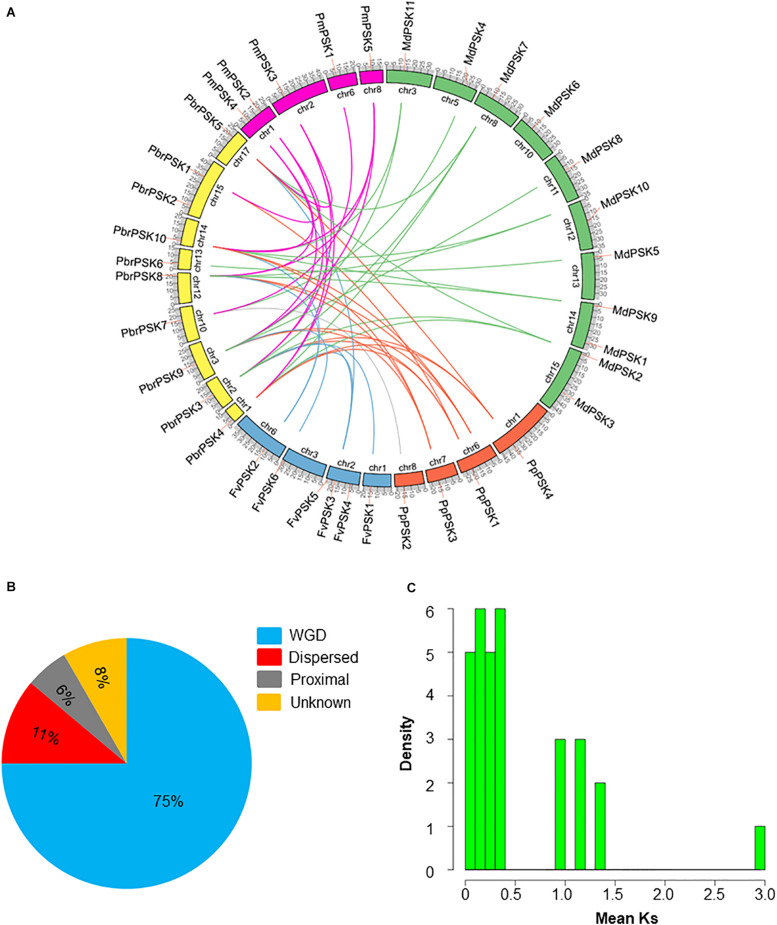
Evolution pattern of PSK genes in pear and other Rosaceae species. **(A)** Synteny analyses of *PbrPSK* genes and other four Rosaceae species. Colorized lines indicate all synteny blocks between pear and other Rosaceae species. Chromosome numbers are indicated on the inner side; gene pairs with syntenic relationships are joined by lines. **(B)** The proportion of duplication model of PSK genes in pear and other Rosaceae. The duplication model includes WGD duplication event, dispersed duplication and proximal duplication. **(C)** Distribution of mean Ks values of *PbrPSK* genes. The *x*-axis represents the mean Ks value; *y*-axis represents the density of the distribution.

Gene duplications play important roles in the expansion of gene families and the generation of new functionalities. To explain the origin of the *PSK* gene family expansion, the MCScanX package was used to analyze the different duplication modes of the *PSK* genes. Three duplication patterns were shown to drive the expansion of the *PSK* gene family: whole-genome, dispersed and proximal duplication. As shown in Supplementary Table S1 and Figure 3B, whole-genome duplication is the main driver of the expansion of the PSK family, as it accounts for 90% (9 for 10), 90% (10 for 11), 75% (3 for 4), and 100% (5 for 5) of gene expansion events studied in pear, apple, peach and Chinese plum, respectively. It is worth noting that two *FvPSKs* (33.3%) were assigned to the proximal duplication block, while the other three (50%) were assigned to the dispersed duplication block. These results showed that a whole-genome duplication event played a critical role in the expansion of the *PSK* gene family in the Rosaceae.

The identification of orthologous genes is required for the accurate understanding of the *PSK* gene family. Therefore, synteny analyses were performed among pear and the other four Rosaceae species. A total of 41 collinear gene pairs were identified among pear and the other Rosaceae species (Figure 3A and Supplementary Table S2). Collinear pairs within the same species were also identified (Supplementary Figure S1) including six pairs in pear, 12 in apple, two in peach and four in Chinese plum. The Ka/Ks ratio for the collinear gene pairs was calculated to identify which selection process drove the evolution of the *PSK* gene family in pear. A ratio of <1 indicates purification selection, a ratio >1 implies positive selection and a ratio = 1 indicates neutral selection. As shown in Supplementary Table S3, all Ka/Ks values were <1 indicating that the PSK gene family was conserved, which is consistent with results seen in *Arabidopsis* (Lorbiecke and Sauter, 2002). The Ks value is usually used to estimate the evolutionary dates of WGD events. Previous studies have shown that the pear genome has undergone two WGD events: a recent WGD (30–45 MYA) and an ancient WGD (∼ 140 MYA) (Wu et al., 2013). According to the Ks value of the *PbrPSK* family in our study, we found that the duplicated gene pairs were distributed at the two Ks value peaks (Figure 3C). Thus, the recent (30–45 MYA) and ancient (∼140 MYA) WGDs might led to the expansion of the *PbrPSK* gene family.

### Expression Profile Analysis of *PSK* Genes in Pear

**FIGURE 4:**
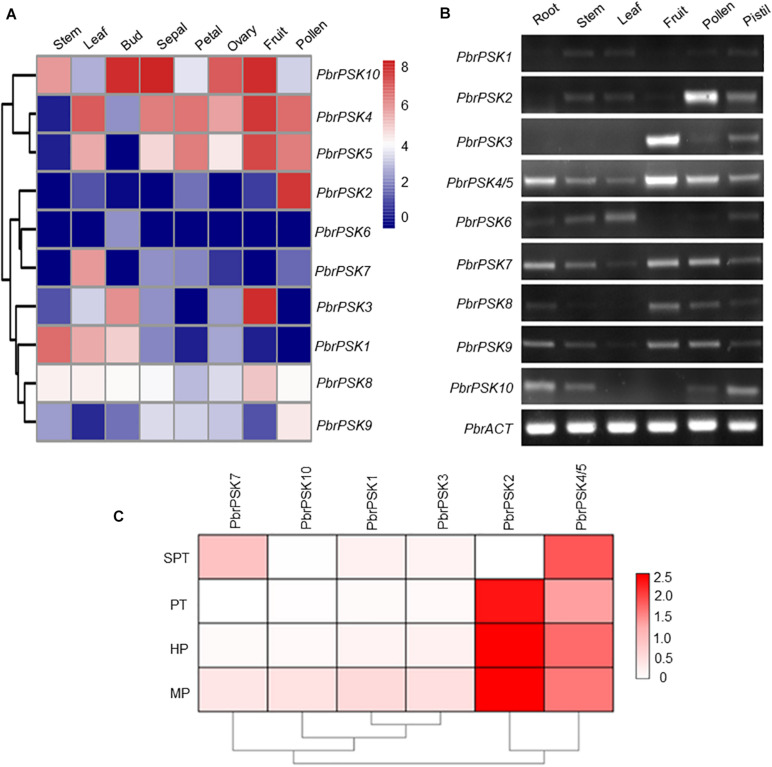
Expression pattern of pear *PSK* genes. **(A)** Expression heatmap of pear *PSK* genes in eight tissues. The expression levels of pear *PSK* genes were measured using RPKM value. Blue indicates a low expression level, white indicates a medium level, and red indicates a high level. **(B)** Expression pattern of *PbrPSK* genes in six tissues using RT-PCR. RT-PCR was performed using root, stem, leaf, fruit, pollen and pistil in pear. An amplified *PbrACT* gene was used as a loading control. Reactions were performed with 30 cycles and experiments were repeated at least three times. **(C)** Heatmap of the expression levels of *PbrPSK* genes during pollen development using qRT-PCR. MP, HP, PT, and SPT correspond to four different developmental stages: mature pollen grains, hydrated pollen, growing pollen tubes 3 h post-hydration and stopped growing pollen tubes, respectively. *PbrTUB* was used as a reference gene.

To study the tissue-specific expression pattern of *PbrPSK* genes in pear, the expression levels of PSK genes in different tissues were determined using transcriptome data (RPKM values) (Figure 4A) and RT-PCR (Figure 4B), however, we focused more on the genes highly expressed in pollen. The results showed that *PbrPSK2* and *PbrPSK4/5* were highly expressed in pear pollen, and *PbrPSK2* shown a highest expression. *PbrPSK2* is highly expressed in pollen, and lowly expressed in multiple tissues (Figures 4A,B). Furthermore, qRT-PCR was used to analyze the expression of *PbrPSK* genes during pollen development in the pear “Dangshansuli.” The following stages were investigated: mature pollen, hydrated pollen, pollen tubes that had been growing for 6 h and pollen tubes that had stopped growing. As shown in Figure 4C, we found that *PbrPSK2* and *PbrPSK4/5* were highly expressed throughout pollen development. Based on the RT-PCR and qRT-PCR results, *PbrPSK2* was selected for the study of its function in pear pollen tube growth.

### PbrPSK2 Regulates Pollen Tube Growth

**FIGURE 5:**
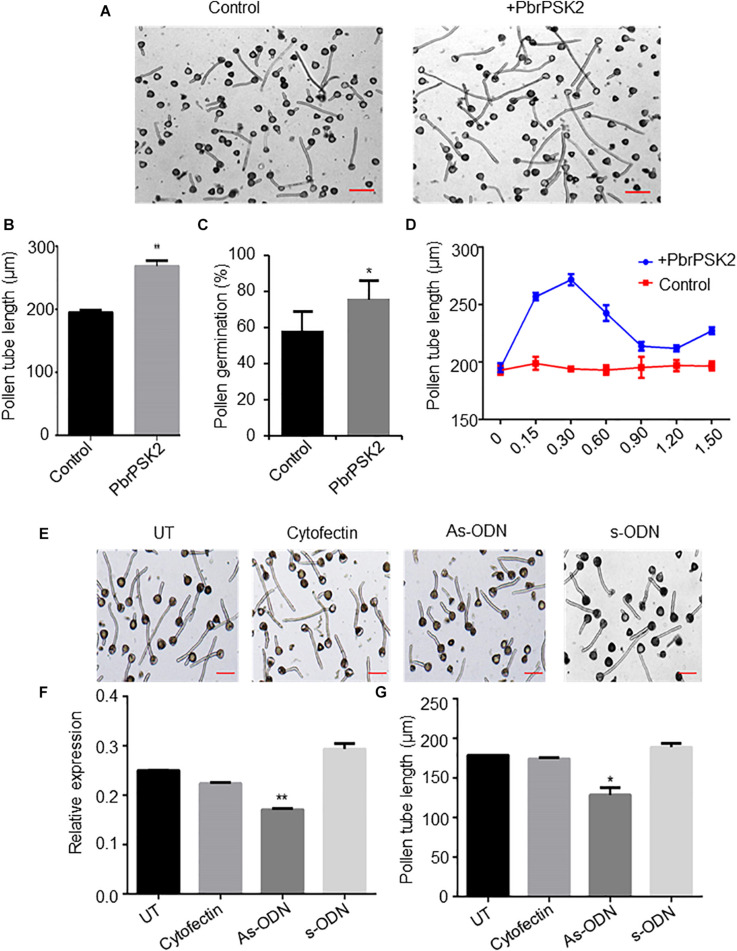
PbrPSK2 promote pear pollen tube growth. **(A)** PbrPSK2 promotes pear pollen tube growth. Images shown were acquired 2 h after treatment without or with purified recombinant PbrPSK2. The experiments were repeated at least three times. Bar = 40 μm. **(B)** The statistical analysis of pollen tube length. Differences were identified using Student’s *t*-test and were considered significant when *p* < 0.01, indicating ^∗∗^. More than one hundred pollen tubes were measured. **(C)** The statistical analysis of pollen germination rate. Differences were identified using Student’s *t*-test and were considered significant when *p* < 0.05, indicating ^∗^. More than one hundred pollen tubes were measured. **(D)** PbrPSK2 promotes pear pollen tube growth in a dose-dependent manner. The statistical analysis of pollen tube length was measured. More than one hundred pollen tubes were measured. **(E)** Knock-down *PbrPSK2* inhibited pollen tube growth using antisense oligonucleotides. UT suggests no treatment controls, cytofectin and s-ODN are used as negative controls. The experiments were repeated at least three times. Bar = 40 μm. **(F)** The expression level of PbrPSK2 is decreased after as-ODN treatment. Cytofection and s-ODN are used as controls. Differences were identified using Student’s *t*-test and were considered significant when *p* < 0.05, indicating ^∗^. **(G)** The statistical analysis of pollen tube length after as-ODN treatment. Differences were identified using Student’s *t*-test and were considered significant when *p* < 0.05, indicating ^∗^. More than one hundred pollen tubes were measured.

To examine the roles of *PbrPSK2* in the growth of pear pollen tubes, we expressed and purified PbrPSK2 in *E. coli* (Supplementary Figure S2) and dialyzed the proteins in pollen medium for 24 h. When we used these purified PbrPSK2 proteins to treat pear pollen, we found that pollen tube growth was significantly promoted (Figures 5A,B). Moreover, pollen germination was significantly promoted by PbrPSK2 (Figure 5C). To check the dose effect of PbrPSK2 on pear pollen tube growth, we used different concentrations of the protein to treat pear pollen. The results showed that when the concentration of PbrPSK2 was 0.3 μmol, the promotion of pollen tube growth was at its strongest, but when the concentration of PbrPSK2 exceeded 0.3 μmol, the promotion of pollen tube growth showed a downward trend, but compared with the control, still had a promoting effect (Figure 5D). These results showed that *PbrPSK2* can promote pear pollen tube growth in a dose-dependent manner.

In addition, to further study the functions of PbrPSK2 on pear pollen tube growth, we used the as-ODN approach to knock down the expression of *PbrPSK2* in pear pollen and used qRT-PCR to detect the subsequent expression level. As shown in Figure 5E, when the expression of *PbrPSK2* was significantly knocked down by ODN treatment (Figure 5F), we observed that the elongation of pollen tubes was significantly inhibited (Figure 5G), indicating PbrPSK2 could promote pear pollen tube growth.

### *PbrPSK2* Increases the Production of ROS in Pear Pollen Tube

**FIGURE 6:**
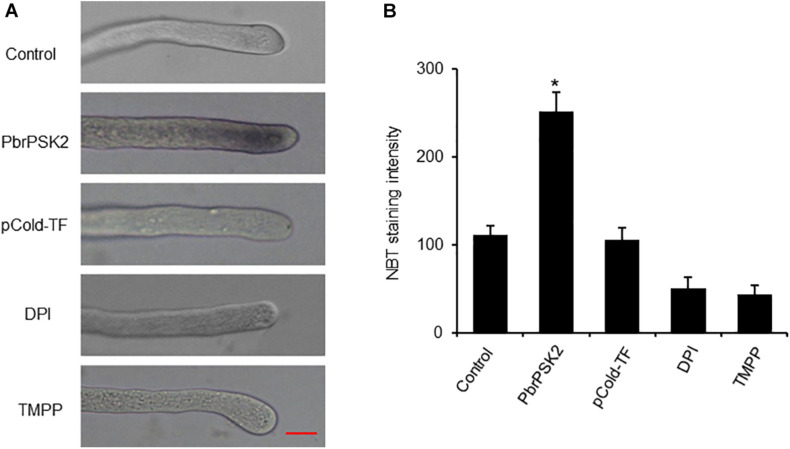
PbrPSK2 induces reactive oxygen species (ROS) production in pollen tubes. **(A)** Typical images of pollen tubes stained with NBT under different treatment conditions for 30 min. At least three independent experiments were repeated with similar results. Mean pixel intensity at the tips of pollen tubes stained with NBT only, NBT with PbrPSK2 (100 nM), NBT with DPI (300 μM), or with Mn-TMPP (300 μM) but without NBT. Bar = 20 μm. **(B)** Statistical analysis of NBT staining intensity in pear pollen tube. Differences were identified using Student’s *t*-test and were considered significant when *p* < 0.05, indicating ^∗^. More than one hundred pollen tubes were measured.

Reactive oxygen species play important roles in pollen tube growth (Potockı et al., 2007; Speranza et al., 2012; Duan et al., 2014). To further study the effect of PbrPSK2, we detected ROS production in pollen tubes using an NBT assay (Wang et al., 2010). NBT can be reduced to blue formazan precipitates by superoxide radicals, so the position of ROS can be detected. Typical images of pollen tubes with NBT staining under different treatments are shown in Figure 6. The control pollen tubes displayed the tip-localized pattern of NBT staining, indicating that pollen tube tips were rich with ROS. Compared with the control and pCold-TF treatment, the gray value at the pollen tube tips increased significantly after PbrPSK2 treatment. ROS at the tips of pollen tubes treated with DPI (a NOX inhibitor) and Mn-TMPP (a ROS scavenger) were removed, and TMPP obviously inhibited pollen tube growth (Supplementary Figure S5), which indicated that ROS are necessary for pear pollen tube growth and these ROS may be produced by an RBOH. When we used PbrPSK2 with TMPP to treat pear pollen, the promotion growth pollen tube was less sensitive to PbrPSK2 (Supplementary Figure S5). These results showed that PbrPSK2 increased the production of ROS and promoted pear pollen tube growth.

## Discussion

Phytosulfokines are plant peptides that play essential roles in many aspects of plant development such as root growth, pollen tube growth, pollen tube guidance and immune responses (Yamakawa et al., 1999; Kutschmar et al., 2009; Stührwohldt et al., 2015). *PSK* gene families have been identified in many plants (Lorbiecke and Sauter, 2002; Stührwohldt et al., 2015). However, the ensuing biological and genetic roles of these peptides in pear and other Rosaceae species remain uncharacterized. In the present work, we performed a comprehensive investigation of the *PSK* gene family in pear and four other Rosaceae species (apple, peach, strawberry and Chinese plum). A total of 10 *PSK* genes were identified in pear, 11 in apple, four in peach, six in strawberry and five in Chinese plum (Table 1). It is interesting that the number of *PSK* genes in pear and apple are almost double the number of in peach, strawberry and Chinese plum. Pear and apple belong to the Maloideae, while strawberry belongs to the Rosoideae, peach and Chinese plum belong to the Prunoideae; a recent whole-genome duplication event occurred in the Maloideae, but not in the Rosoideae or Prunoideae. Therefore, the results indicated that the recent whole-genome duplication may have led to the expansion of the PSK gene family in the Maloideae.

Phylogenetic relationship can provide novel insights into the evolution of diverse gene family members and gene multiplicity (Smith et al., 2008). The comparative phylogenetic analysis in this study showed that *PSK* genes in pear and other Rosaceae species can be categorized into three distinct subclasses, which is similar to the classification of PSK genes in *Arabidopsis* (Figure 1). Gene structural diversity can offer important information on the evolutionary history of gene families, providing additional help to phylogenetic classification (Ames et al., 2012). Based on the phylogenetic analysis, PSKs with similar motif compositions and gene structures were clustered, which were similar to results described for *Arabidopsis*. These findings suggest that PSK genes are highly conserved.

Gene duplication, which may occur through chromosomal segmental duplication or tandem duplication, is thought to be an important means of expanding and gaining functional diversity during evolution (Schacherer et al., 2005; Suhre, 2005). Whole-genome, tandem and segmental duplication are the three main duplication models for gene family expansion (Freeling, 2009). In this study, analysis of duplication models showed that most *PSK* genes in pear and other Rosaceae species were duplicated in WGD duplication events (Figure 3B and Supplementary Table S1). The number of homologous genes between pear and apple considerably exceeds the number of homologous genes between pear and the other Rosaceae species studied. This result may be due to the recent whole-genome duplication event in pear, which is consistent with the two Ks value peaks in *PbrPSK* duplicated gene pairs, which indicated that the recent (30–45 MYA) and ancient (∼140 MYA) WGDs led to the expansion of *PSK* genes in pear. The Ka/Ks ratios were used to represent the selection constraint for evolutionary selection (Hu and Banzhaf, 2008). Our result showed that all the ratios of duplicated pairs in *PSK* genes were <1 (Supplementary Table S3), indicating that the *PSK* genes were conserved.

Gene expression patterns provide important information on gene function. In *Arabidopsis*, many members of the PSK family have been reported to be involved in cell development (Yamakawa et al., 1998; Yang et al., 1999; Kim et al., 2006). To dissect the expression patterns of *PSK* genes in pear, Transcriptome data (RPKM values) and RT-PCR were used to map the expression level in different tissues of pear. For the *PSK* genes in pear, we were most interested in those which play important roles during pear reproductive processes. The results showed that five *PSK* genes were expressed in pear pollen (*PbrPSK2*, *PbrPSK4/5*, *PbrPSK7*, *PbrPSK8*, and *PbrPSK9*), with *PbrPSK2* showing the largest expression level (Figures 4A,B). *PbrPSK2* was expressed at a low level in multiple tissues, but highly expressed in pollen, indicating that *PbrPSK2* may play important roles in pear pollen development. Furthermore, we examined the expression level in pear pollen tube development using qRT-PCR and generated a heatmap (Figure 4C). Based on the heatmap, *PbrPSK4/5* was expressed in all four stages of pollen tube growth. *PbrPSK2* was highly expressed at all stages of pollen tube development except in those that had stopped growth. The expression level of *PbrPSK2* in pollen tube growth was significantly higher than that for other *PbrPSK* genes. These results further demonstrated that *PbrPSK2* is likely to play important roles in pear pollen tube growth.

Many proteins have been identified as regulators in pollen tube growth. For example, pollen tube growth in *Arabidopsis* and tomato is respectively inhibited by AtRALF4/19 and SlRALF (Covey et al., 2010; Mecchia et al., 2017). Transmitting-tract-specific protein (TTS), as positive regulator, plays important roles in pollen tube growth (Wu et al., 2000). In this study, several pieces of evidence suggested a promoting effect of PbrPSK2 on pear pollen tube growth. In pear pollen treated with purified *E. coli*-expressed PbrPSK2, pollen tube elongation was promoted in a dose-dependent manner (Figures 5A–D). When the expression level of *PbrPSK2* was knocked down in pear pollen using ODN, the pollen tube grew slower (Figures 5E–G). These results provide more evidence that pollen peptides, which pollen itself produces, regulate pollen tube growth.

Reactive oxygen species production is essential for cell signaling and regulation, but excess ROS accumulation is injurious to cell survival (Thannickal and Fanburg, 2000). Excessive ROS in cells can serve as a second messenger to mediate multiple signaling pathways (Bae et al., 2011). In our study, we examined the ROS production of pollen tubes after PbrPSK2 treatment using NBT. We observed that PbrPSK2 could increase the production of ROS in pear pollen tubes (Figure 6). Its possible mechanism is that PbrPSK2 activates downstream receptors that transduce signals into the cytosol, leading to the production of ROS and elongation of pollen tubes. In summary, based on our results, PbrPSK2 may promote pollen tube growth, but its mechanism for regulating pollen tube growth requires further study.

## Data Availability Statement

The raw data supporting the conclusions of this article will be made available by the authors, without undue reservation, to any qualified researcher.

## Author Contributions

XK and QL conceived the experimental design. XK performed experiments and data analyses. QL and YS contributed synteny analyses and the Perl script, and configured some of the figures. PW revised the final manuscript. JW and SZ managed the experiments. All authors read and approved the final manuscript.

## Conflict of Interest

The authors declare that the research was conducted in the absence of any commercial or financial relationships that could be construed as a potential conflict of interest.

## Footnotes

1 http://pfam.xfam.org/family/PF06404

2 http://www.rosaceae.org/

3 http://www.phytozome.net/search.php

4 http://peargenome.njau.edu.cn/

5 http://prunusmumegenome.bjfu.edu.cn/index.jsp

6 http://smart.embl-heidelberg.de/

7 http://web.expasy.org/compute_pi/

8 http://www.clustal.org/

9 http://www.bioinformatics.org/sms2/

10 http://pfam.xfam.org/family/PF06404

11 http://www.evolgenius.info/evolview/

12 http://meme.nbcr.net/meme/cgibin/meme.cgi

13 http://circos.ca/

14 http://rna.tbi.univie.ac.at/cgi-bin/RNAWebSuite/RNAfold.cgi

## Supplementary Material

Supplementary Figure 1 Synteny analyses of PSK genes in the same species and chromosome location. Chromosome numbers are indicated on the inner side; gene pairs with syntenic relationships are joined by lines.

Supplementary Figure 2 PbrPSK2 protein expression and purification *in vitro*. Recombinant proteins for the PbrPSK2 and purified using the *E. coli* system.

Supplementary Figure 3 Alignment of PbrPSK4 and PbrPSK5 amino acid sequences. PbrPSK4 and PbrPSK5 shared 100% identity at the amino acid level.

Supplementary Figure 4 The multiple sequence alignments from all identified PSK homologs and some functionally characterized PSKs.

Supplementary Figure 5 ROS are necessary for pear pollen tube growth. **(A)** The ROS effect on pollen tube growth. The ROS scavenger TMPP arrests pollen tube elongation. Images shown were acquired 2 h after treatment PbrPSK2, TMPP or PbrPSK2 with TMPP. The experiments were repeated at least three times. Bar = 40 μm. **(B)** The statistical analysis of pollen tube length was measured. More than one hundred pollen tubes were measured.

Supplementary Table 1 Numbers of PSK genes from different origins in five Rosaceae genomes.

Supplementary Table 2 The orthology of PSK genes in five Rosaceae species.

Supplementary Table 3 Ka and Ks substitution rates between gene pairs in pear.

Supplementary Table 4 Primers of PbrPSK genes for this work (^∗^represents the modification).
