# Supplementary figures and images for: The Peptide PbrPSK2 From Phytosulfokine Family Induces Reactive Oxygen Species (ROS) Production to Regulate Pear Pollen Tube Growth
Source: Front Plant Sci. 2020 Nov 30;11:601993. doi: 10.3389/fpls.2020.601993 (PMC7734187; doi:10.3389/fpls.2020.601993)

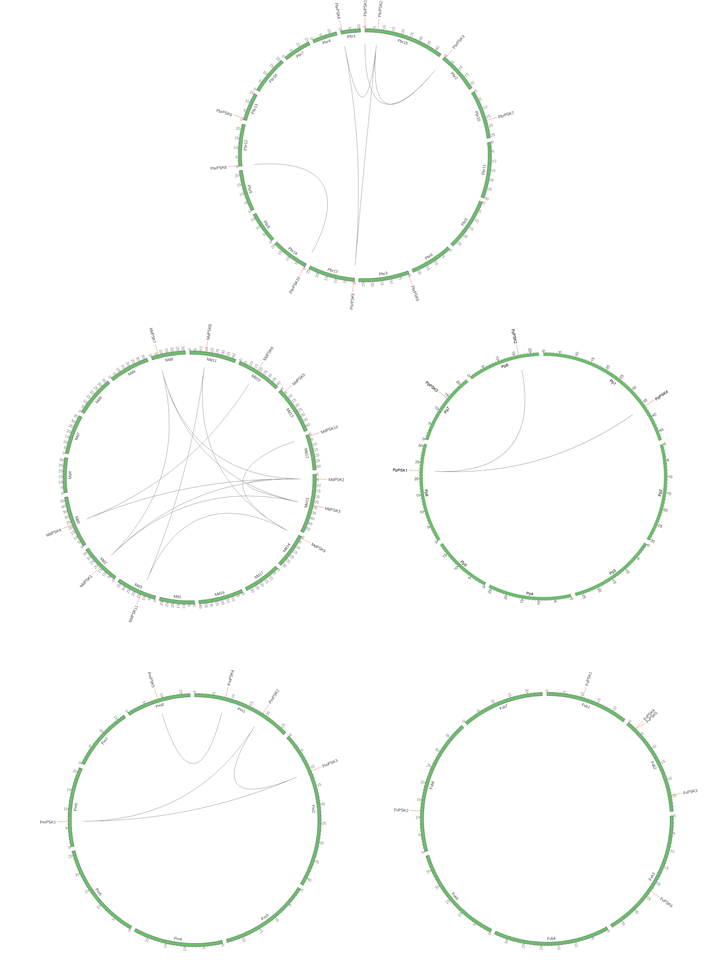

Supplement: Supplementary Figure 1 — Synteny analyses of PSK genes in the same species and chromosome location. Chromosome numbers are indicated on the inner side; gene pairs with syntenic relationships are joined by lines. [file Image_1.TIF]

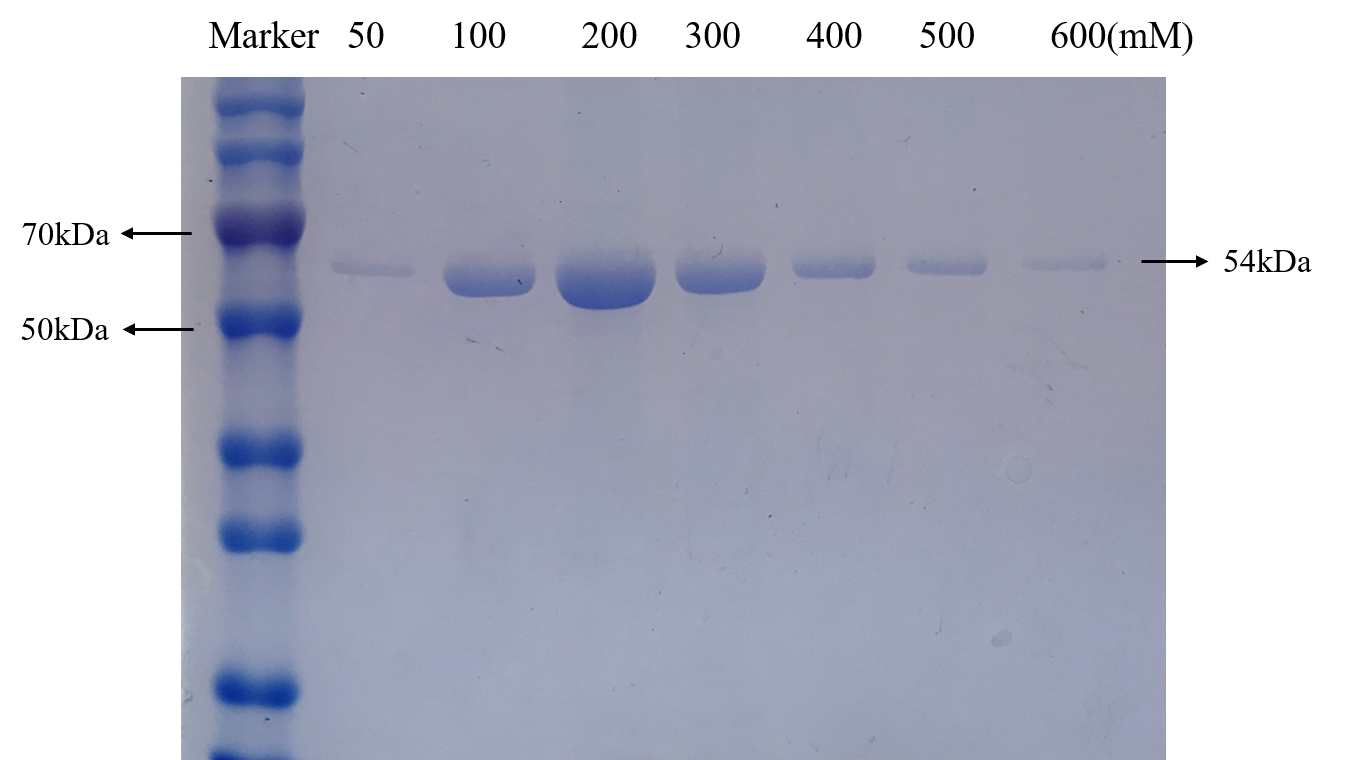

Supplement: Supplementary Figure 2 — PbrPSK2 protein expression and purification in vitro. Recombinant proteins for the PbrPSK2 and purified using the E. coli system. [file Image_2.TIF]

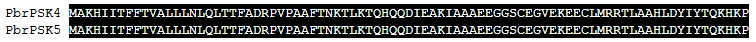

Supplement: Supplementary Figure 3 — Alignment of PbrPSK4 and PbrPSK5 amino acid sequences. PbrPSK4 and PbrPSK5 shared 100% identity at the amino acid level. [file Image_3.TIF]

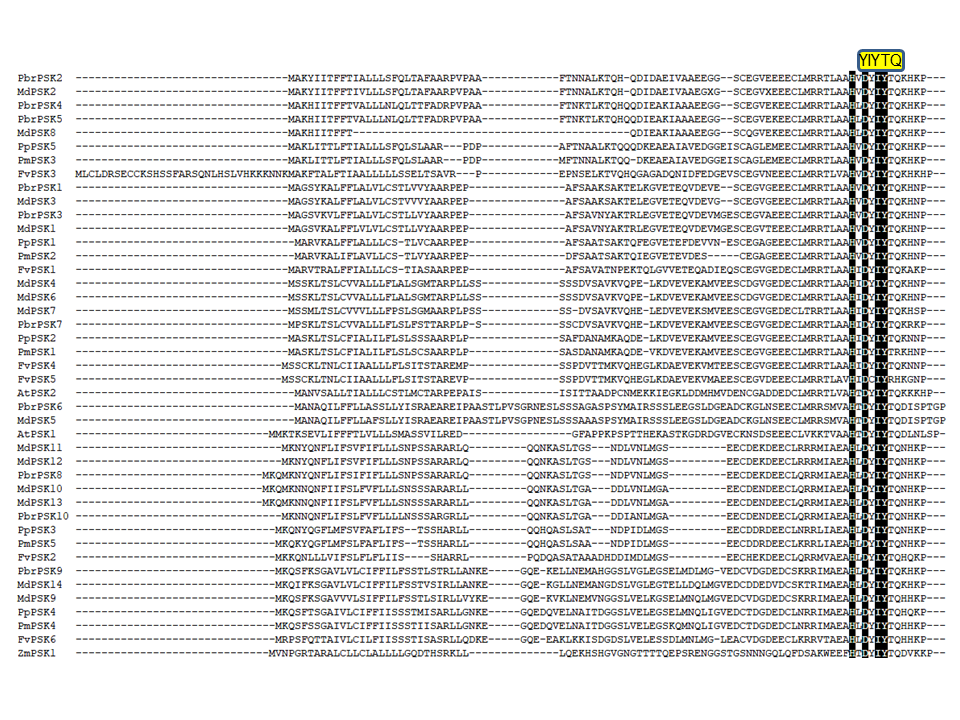

Supplement: Supplementary Figure 4 — The multiple sequence alignments from all identified PSK homologs and some functionally characterized PSKs. [file Image_4.TIF]

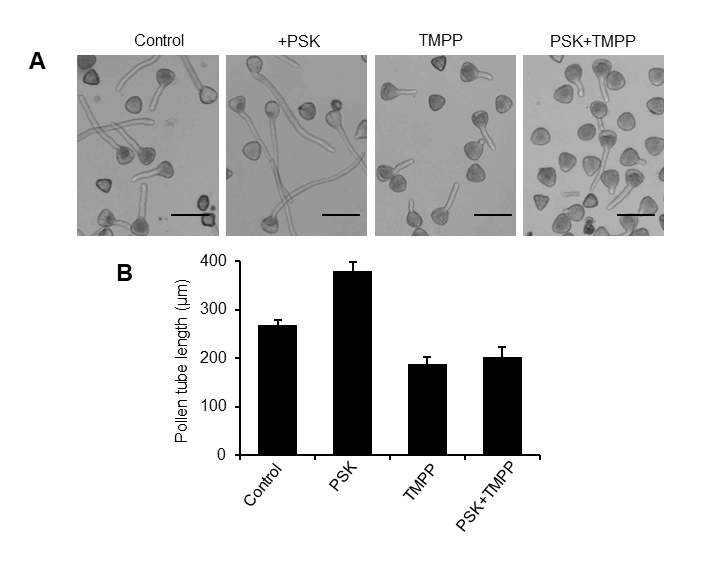

Supplement: Supplementary Figure 5 — ROS are necessary for pear pollen tube growth. (A) The ROS effect on pollen tube growth. The ROS scavenger TMPP arrests pollen tube elongation. Images shown were acquired 2 h after treatment PbrPSK2, TMPP or PbrPSK2 with TMPP. The experiments were repeated at least three times. Bar = 40 μm. (B) The statistical analysis of pollen tube length was measured. More than one hundred pollen tubes were measured. [file Image_5.TIF]
